# Regulatory T Cells Suppress Inflammation and Blistering in Pemphigoid Diseases

**DOI:** 10.3389/fimmu.2017.01628

**Published:** 2017-11-24

**Authors:** Katja Bieber, Shijie Sun, Mareike Witte, Anika Kasprick, Foteini Beltsiou, Martina Behnen, Tamás Laskay, Franziska S. Schulze, Elena Pipi, Niklas Reichhelm, René Pagel, Detlef Zillikens, Enno Schmidt, Tim Sparwasser, Kathrin Kalies, Ralf J. Ludwig

**Affiliations:** ^1^Lübeck Institute of Experimental Dermatology, University of Lübeck, Lübeck, Germany; ^2^Department of Dermatology, University of Lübeck, Lübeck, Germany; ^3^Department for Infectious Diseases and Microbiology, University of Lübeck, Lübeck, Germany; ^4^Institute of Anatomy, University of Lübeck, Lübeck, Germany; ^5^Institute for Experimental Infection Research, TWINCORE, Centre for Experimental and Clinical Infection Research, A Joint Venture between the Helmholtz Centre for Infection Research and the Hannover Medical School, Hanover, Germany

**Keywords:** regulatory T cells, autoimmunity, skin, pemphigoid disease, neutrophil activation, Th1, Th2

## Abstract

Regulatory T cells (Tregs) are well known for their modulatory functions in adaptive immunity. Through regulation of T cell functions, Tregs have also been demonstrated to indirectly curb myeloid cell-driven inflammation. However, direct effects of Tregs on myeloid cell functions are insufficiently characterized, especially in the context of myeloid cell-mediated diseases, such as pemphigoid diseases (PDs). PDs are caused by autoantibodies targeting structural proteins of the skin. Autoantibody binding triggers myeloid cell activation through specific activation of Fc gamma receptors, leading to skin inflammation and subepidermal blistering. Here, we used mouse models to address the potential contribution of Tregs to PD pathogenesis *in vivo*. Depletion of Tregs induced excessive inflammation and blistering both clinically and histologically in two different PD mouse models. Of note, in the skin of Treg-depleted mice with PD, we detected increased expression of different cytokines, including Th2-specific *IL-4, IL-10*, and *IL-13* as well as pro-inflammatory Th1 cytokine *IFN-γ* and the T cell chemoattractant *CXCL-9*. We next aimed to determine whether Tregs alter the migratory behavior of myeloid cells, dampen immune complex (IC)-induced myeloid cell activation, or both. *In vitro* experiments demonstrated that co-incubation of IC-activated myeloid cells with Tregs had no impact on the release of reactive oxygen species (ROS) but downregulated β2 integrin expression. Hence, Tregs mitigate PD by altering the migratory capabilities of myeloid cells rather than their release of ROS. Modulating cytokine expression by administering an excess of IL-10 or blocking IFN-γ may be used in clinical translation of these findings.

## Introduction

Regulatory T cells (Tregs) are of major importance in modulating host responses to tumors and infections and in inhibiting the development of autoimmunity and allergies mostly through regulating adaptive immune functions. The effects of Tregs on adaptive immune functions are well characterized (1). Evidence also supports the notion that Tregs can indirectly, through the modulation of antigen-specific T cells, dampen myeloid cell-driven immune responses (2). However, if and how Tregs can directly modulate myeloid cell-dependent inflammation has been less studied. The role of Tregs in skin inflammation has previously been shown. The percentage of Tregs in skin infiltrate is considerable since in humans, 5–20% of resident T cells in the skin are Tregs (3), and in mice, the percentage is even higher (60–80%) (4). Thus, the skin, an outermost organ constantly exposed to external insults, appears to serve as a major site for the immunosuppressive action of Tregs. Reducing the number of Tregs in neonatal mice leads to the development of a scurfy-like disease in “depletion of regulatory T cell” (DEREG) mice. By contrast, the depletion of Tregs in adult mice is not sufficient to induce clinical symptoms (5), but Treg-deficient scurfy mice bear an autoimmune phenotype (6).

Further studies have attempted to unravel the mechanism by which Tregs modulate neutrophil functions. In a mouse melanoma model, Tregs limit neutrophil accumulation and survival. This effect is associated with decreased expression of the neutrophil chemoattractants CXCL1 and CXCL2, which promote the survival of inoculated tumor cells (7). Further *in vitro* coculture assays using LPS-stimulated human Tregs and neutrophils showed a decrease in CD62L shedding after 45 min of incubation and a decrease in IL-6, IL-8, and TNF-α production after 18 h of incubation. Neutrophil death was accelerated doubly in the presence of Tregs that had been stimulated with LPS (8). Currently, there is a knowledge gap concerning the influence of Tregs on immune complex (IC)-stimulated inflammation.

Prototypical IC-dependent diseases are pemphigoid diseases (PDs). Here, skin inflammation and subepidermal blistering are caused by autoantibodies directed against structural proteins. However, in most PDs, autoantibody binding alone is not sufficient to cause clinical disease manifestation. For the latter, myeloid cells are a prerequisite. By activating specific Fc gamma receptors, myeloid cells bind to skin-bound ICs, get activated and ultimately release reactive oxygen species (ROS) and proteases, leading to inflammation and blistering (9–12). The involvement of macrophages/monocytes was shown in *ex vivo* assays of human skin (13), but not *in vivo*. Regarding cell types besides myeloid cells, mast cells are required to induce the PD bullous pemphigoid [BP, mediated by autoantibodies against type XVII collagen (COL17)] (14), while in the PD epidermolysis bullosa acquisita [EBA, mediated by autoantibodies against type VII collagen (COL7)], mast cells were activated, but dispensable for inflammation and blistering (15). Of note, in these antibody transfer-induced models of PD, a role of natural killer T (NKT) cells and γδ T cells was recently shown; both cell types are able to increase CD18 expression and CD62L shedding in neutrophils, thereby contributing to a more inflammatory phenotype by releasing TNFα (16). The involvement of other T cells in the effector phase of PDs has not been shown yet. Interestingly, in BP patients, alterations in the T cell compartment have been noted. Specifically, compared with healthy controls or patients with pemphigus, BP patients had lower Treg numbers in the skin and circulation, while Th17 cells were found more frequently in the skin of these patients (17, 18). PD animal models allow distinguishing between effects of these cells on tolerance loss or autoantibody production and events leading to skin inflammation and blistering (19). For this reason, we used antibody transfer-induced disease models for the investigation of the role of Tregs during IC-induced inflammation.

## Materials and Methods

### Mice

C57BL/6J mice were obtained from Charles River Laboratories (Sulzfeld, Germany). FoxP3^DTR-eGFP^ (DEREG) mice were kindly provided by Tim Sparwasser, Hannover, Germany (5). Heterozygous DEREG mice were set up for mating with wild-type littermates. Gender-matched littermates aged 8–12 weeks were used for the experiments. The mice were fed with standardized mouse chow and acidified drinking water *ad libitum*. All clinical examinations, biopsies and bleedings were performed under anesthesia using intraperitoneal (i.p.) administration of a mixture of ketamine (100 mg/g, Sigma-Aldrich, Taufkirchen, Germany) and xylazine (15 mg/g, Sigma-Aldrich). All animal experiments were conducted according to the European Community rules for animal care, approved by the respective governmental administration [V242.29833/2016(49-4/16), V245-46582/2015(78-5/12), V312-7224.122-5(30-2/13)], Ministry for Energy, Agriculture, the Environment and Rural Areas and performed by certified personnel.

### Generation of Anti-Mouse COL7 and Anti-COL17 IgG

Total rabbit anti-mouse COL7 IgG and rabbit anti-mouse COL17 IgG were prepared as previously described (16, 20). Rabbits were immunized with recombinant proteins of the non-collagenous (NC)-1 domain of murine COL7 or the extracellular portion of murine COL17 (NC15A), which were supplied commercially (Eurogentec, Seraing, Belgium). IgG from immune and normal rabbit sera was purified by affinity chromatography using protein G. The reactivity of all IgG fractions was analyzed by immunofluorescence microscopy of murine skin.

### Induction of Experimental EBA and BP in DEREG Mice

Antibody transfer-induced studies for the induction of experimental EBA and BP followed published protocols with minor modifications (16, 20). To induce Treg depletion, mice were treated with 1 µg diphtheria toxin (DT, Sigma-Aldrich)/100 μl PBS/mouse on days 1, 2, 5, 8, and 11 after initial IgG injections. For *experimental EBA induction*, mice received a total of four i.p. injections of 1 mg rabbit anti-mCOL7 IgG on days 0, 3, 6, and 9 (in total 4 mg rabbit anti-mCOL7 IgG). For *experimental BP induction*, mice received a total of six i.p. injections of 5 mg rabbit anti-mCOL17 IgG on days 0, 2, 4, 6, 8, and 10 (in total 30 mg rabbit anti-mCOL17 IgG). Different body parts were individually scored by the appearance of crust, erythema, lesions and/or alopecia by blinded personal. Control animals were injected with a total of four i.p. injections of 1 mg normal rabbit IgG on days 0, 3, 6, and 9 (in total 4 mg IgG). The total body score is a composite of 2.5% per ear, snout and oral mucosa; 0.5% per eye; 9% for head and neck (excluding eyes, ears, oral mucosa, and snout); 5% per front limb; 10% per hind limb and tail; and 40% for the remaining trunk (21). Blood and tissue samples were collected on days 5 and 12. Serum was collected from the blood samples by centrifugation and was analyzed for cytokine release using the LEGENDplex™ Mouse Inflammation Panel (BioLegend) as described by the manufacturer’s protocol. Tissue samples were snap frozen in liquid nitrogen for the analysis of mRNA and for immunostainings.

All experiments were repeated with a minimum of two independent experiments using different batches of purified IgG.

### Immunofluorescence, Immunohistochemistry, and Histological Studies

Direct *immunofluorescence* microscopy was performed to detect rabbit IgG and murine C3 in experimental PD as described (16, 20). Briefly, frozen sections were prepared from tissue biopsies and incubated with FITC-labeled goat anti-rabbit IgG antibody (Dako Deutschland GmbH, Hamburg, Germany). For *histology*, skin samples were fixed in 3.7% paraformaldehyde. The 8-μm-thick sections from paraffin-embedded tissues were stained with hematoxylin and eosin (H&E) according to standard protocols. For *immunohistochemistry*, paraffin sections from lesional skin were stained for T cells and granulocytes as previously described (16). Briefly, mAbs against CD4 (BD Biosciences, Heidelberg, Germany) and Gr-1 (BD Biosciences) were used as primary antibodies, and biotin rabbit anti-rat IgG (Dako) was used as the secondary Ab, followed by detection with ExtrAvidin^®^ alkaline phosphatase (Sigma-Aldrich). Alkaline phosphate activity was visualized with Fast Blue (BB Salt, Sigma-Aldrich). Samples were stained by hematoxylin according to standard protocols.

### Flow Cytometry

For FACS analysis, the following antibodies were used: Vio-Green; Brilliant Violet 421™; FITC-, Alexa 647-, PE-, allophycocyanin (APC)-, or APC-Vio770-conjugated anti-mouse CD4 (clone L3T4, or RM4-5); Gr-1 (clone RB6-8C5); CD45 (clone 30F11); CD18 (clone M18/2); CD62L (clone MEL-14); CD11b (clone M1/70); CD25 (clone PC61); Ly6G (clone 1A8); Foxp3 (clone MF23); or appropriate isotype control antibodies (eBioscience *via* Thermo Fisher Scientific, Dreieich, Germany, Miltenyi Biotec, Bergisch-Gladbach, Germany or BD). After erythrocyte lysis cell suspensions were blocked with anti-mouse CD16/CD32 mAb before staining, and dead cells were excluded from the analysis using propidium iodide (PI). Briefly, for the staining of CD45/Gr-1/CD11b and CD45/CD4 cells, blocked single cell suspensions from spleen and blood of mice suffering from experimental PD were first gated for singlets (FSC-H compared with FSC-A) and leukocytes (SSC-A compared with FSC-A). The leukocyte gates were further analyzed for their uptake of PI to differentiate between live and dead cells and for their expression of CD45, thus, selecting only the live, healthy leukocyte population. To further analyze the purity of isolated Tregs and PMNs for *in vitro* analysis, cells were stained with CD4/Foxp3/CD25 or Ly6G/CD45/PI, respectively. For PMNs, the purity and viability was ≥90%; for Tregs, the purity was 80%. To determine the activation status of PMNs after treatment w/o ICs and Tregs, the cells were stained with CD45/CD62L/CD18/Ly6G/PI. All stainings were performed using standard protocols for cell surface staining, except for CD4/Foxp3/CD25, where intranuclear staining was performed using FOXP3 Fix/Perm Buffer (BioLegend, San Diego, CA, USA) and BD Perm/Wash™ buffer following manufacturer protocols. FACS analysis was performed using Miltenyi MacsQuant10 or FACSCalibur with MACSQuantify™ (version 2.8) or BD CellQuest Pro (version 5.1) software.

### Assessment of Neutrophil Activation by Analysis of Cell Surface Markers and Cytokine Release

PMNs were isolated from the femurs and tibias of healthy C57BL/6J mice as described in detail elsewhere (16). Tregs were isolated from the spleen of the same animal using a CD4^+^CD25^+^ Regulatory T Cell Isolation Kit, mouse (Miltenyi) following the manufacturer’s protocol. The enrichment of cells was determined by FACS. In total, 2 × 10^5^ PMNs/100 μl were stimulated with ICs formed by 10 µg/ml mCOL7 and 2 µg/ml rabbit anti-mCOL7 IgG as described elsewhere (22) for 60 min at 37°C. Isolated Tregs were then cocultured with the IC-stimulated PMNs for an additional 4.5 h in a ratio of 1:4 (5 × 10^4^ Tregs/2 × 10^5^ PMN/200 μl). To evaluate the activation status, cells were stained for flow cytometry analysis using CD18-FITC, CD62L-PE-Vio770, Ly6G-APC-Vio770, and CD45-VioGreen (Miltenyi) following standard procedures. Dead cells were excluded using PI.

### Assessment of Neutrophil Activation by ROS

Neutrophil activation was assessed by determining IC-induced ROS release using a previously published protocol (16). Isolated murine neutrophils (2 × 10^5^ cells/100 μl) were preincubated w/isolated murine Tregs (5 × 10^4^ cells/200 μl), for 1 h at 37°C (without ICs), followed by incubation on a 96-well plate (Lumitrac 600, Greiner Bio-One, Frickenhausen, Germany) coated with ICs formed by 10 µg/ml mCOL7 and 2 µg/ml rabbit anti-mCOL7 IgG. ROS release was analyzed using luminol (Sigma-Aldrich) (22). Each plate was analyzed for 99 repeats using a plate reader (GloMax^®^-Multi Detection System, Promega GmbH, Mannheim, Germany); the values are expressed as relative luminescence units.

### Assessment of Neutrophil Activation by NETosis

Neutrophil activation was assessed by determining neutrophil extracellular trap (NET) formation using a previously published protocol (16, 23). Blood collection was conducted with the understanding and written consent of each participant and was approved by the Ethical Committee of the Medical Faculty of the University of Lübeck (09-140). Isolated human neutrophils (2 × 10^5^ cells) were either stimulated with 20 nM PMA or with ICs formed by 10 µg/ml mCOL7 and 2 µg/ml rabbit anti-mCOL7 IgG. The stimulation was performed w/o isolated human Tregs (5 × 10^4^ cells) on a 96-well FLUOTRAC™ 600 plate (Greiner Bio-One) in 10 mM HEPES-buffered medium. NET formation was analyzed for 7 h (IC) or 4 h (PMA) every 5 min at 37°C by using Tecan infinite M200 Pro reader and Tecan i-control 1.7 Software. CO_2_ control was achieved during the assay by the use of a Tecan gas module. For statistical analysis, the area under the curve was calculated.

### RNA Extraction, Reverse Transcription, and Real-time Quantitative PCR

For gene expression analysis in skin sections, 10 cryo-sections (12 µm) were prepared and used for RNA isolation, reverse transcription, and real-time RT-PCR as previously described (24). Briefly, total RNA was isolated according the manufacturer’s protocol (innuPrep RNA Mini Kit, Analytic Jena AG). After reverse transcription, the cDNA was added to either qPCR Master Mix Plus or qPCR Master Mix SYBR Green Plus (Thermo Fisher Scientific Inc., Waltham, USA) and amplified using an SDS ABI 7900 system (Applied Biosystems, Darmstadt, Germany). The amount of cDNA copies was normalized to the housekeeping gene GAPDH using the 2^ΔCT method.

Primer sequences and concentrations of the analyzed genes were previously published (24, 25) or depicted below: *Ccr5* (for: 5′-CCC ACT CTA CTC CCT GGT ATT C-3′; rev: 5′-GCA GGA AGA GCA GGT CAG AG-3′; 0.5 µM each), *Ccr7* (for: 5′-TGG TGG TGG CTC TCC TTG-3′; rev: 5′-GGC CTT AAA GTT CCG CAC ATC-3′; 0.5 µM each), *Cd11c* (for: 5′-CCA CTG TCT GCC TTC ATA TTC-3′; rev: 5′-GAC GGC CAT GGT CTA GAG-3′; 0.5 µM each), Cd19 (for: 5′-GAA AAT GCA GAT GAG GAG CTG G-3′; rev: 5′-GCT GCA TAG AGG ATC CCT CTC-3′; probe: 5′-CAA CCA GTT GGC AGG ATG ATG GAC TTC CT-3′), *Cd3* (for: 5′-ATA GGA AGG CCA AGG CCA AG-3′; rev: 5′-TCA GGC CAG AAT ACA GGT C-3′; probe: 5′-CCA GAC TAT GAG CCC ATC CGC AAA GG-3′), *Cxcl-1/KC* (for: 5′-CAG ACC ATG GCT GGG ATT C-3′; rev: 5′-GAA CCA AGG GAG CTT CAG-3′; probe: 5′-CCT CGC GAC CAT TCT TGA GTG TGG CTA TGA C-3′), *Cxcl-10/IP-10* (for: 5′-GAG GGC CAT AGG GAA GCT TG-3′; rev: 5′-CGG ATT CAG ACA TCT CTG CTC-3′; probe: 5′-CAT CGT GGC AAT GAT CTC AAC ACG TGG-3′), *Cxcl-2/MIP-2* (for: 5′-AGT GAA CTG CGC TGT CAA TG-3′; rev: 5′-GCT TCA GGG TCA AGG CAA AC-3′; 0.125 µM each), *Cxcl-9/MIG* (for: 5′-TTG GGC ATC ATC TTC CTG GAG-3′; rev: 5′-GCA GGA GCA TCG TGC ATT C-3′; probe: 5′-CTT ATC ACT AGG GTT CCT CGA ACT CCA CAC-3′), *Gapdh* (for: 5′-GAC GGC CGC ATC TTC TTG T-3′; rev: 5′-CAC ACC GAC CTT CAC CAT TTT-3′; probe: 5′-CAG TGC CAG CCT CGT CCC GTA GA-3′), Gr-1 (for: 5′-GCG TTG CTC TGG AGA TAG AAG-3′; rev: 5′-CTT CAC GTT GAC AGC ATT ACC-3′; 0.25 µM each), *Ifn-g* (for: 5′-GCA AGG CGA AAA AGG ATG C-3′; rev: 5′-GAC CAC TCG GAT GAG CTC ATT G-3′; probe: 5′-TGC CAA GTT TGA GGT CAA CAA CCC ACA G-3′), *Il-10* (for: 5′-TCC CTG GGT GAG AAG CTG AAG-3′; rev: 5′-CAC CTG CTC CAC TGC CTT G-3′; probe: 5′-CTG AGG CGC TGT CAT CGA TTT CTC CC-3′), *Il-13* (for: 5′-GGA GCT TAT TGA GGA GCT GAG-3′; rev: 5′-CAG GGA ATC CAG GGC TAC AC-3′; probe: 5′-CAT CAC ACA AGA CCA GAC TCC CCT GTG C-3′), *Il-17a* (for: 5′-TCA GAC TAC CTC AAC CGT TCC-3′; rev: 5′-CTT TCC CTC CGC ATT GAC AC-3′; probe: 5′-CAC CCT GGA CTC TCC ACC GCA ATG AAG-3′), *Il-33* (for: 5′-GTG ATC AAT GTT GAC GAC TCT GG-3′; rev: 5′-GGG ACT CAT GTT CAC CAT CAG-3′; 0.5 µM each), *Il-4* (for: 5′-GAG ACT CTT TCG GGC TTT TCG-3′; rev: 5′-AGG CTT TCC AGG AAG TCT TTC AG-3′; probe: 5′-CCT GGA TTC ATC GAT AAG CTG CAC CAT G-3′), *Itgam/Mac-1* (for: 5′-CTT CAC GGC TTC AGA GAT GAC-3′; rev: 5′-CTG AAC AGG GAT CCA GAA GAC-3′; 0.5 µM each), *Tnf-a* (for: 5′-CCC TCA CAC TCA GAT CAT CTT CTC-3′; rev: 5′-TGG CTC AGC CAC TCC AG-3′; probe: 5′-CTG TAG CCC ACG TCG TAG CAA ACC AC-3′).

### Statistical Analysis

The data were analyzed using SigmaPlot, version 12 (Systat Software Inc., Chicago, IL, USA). Applied tests and confidence intervals are indicated in the respective text and figure legends. A *p*-value < 0.05 was considered statistically significant.

## Results

### Depletion of Tregs Induces Excessive Disease Progression during PD

**Figure 1:**
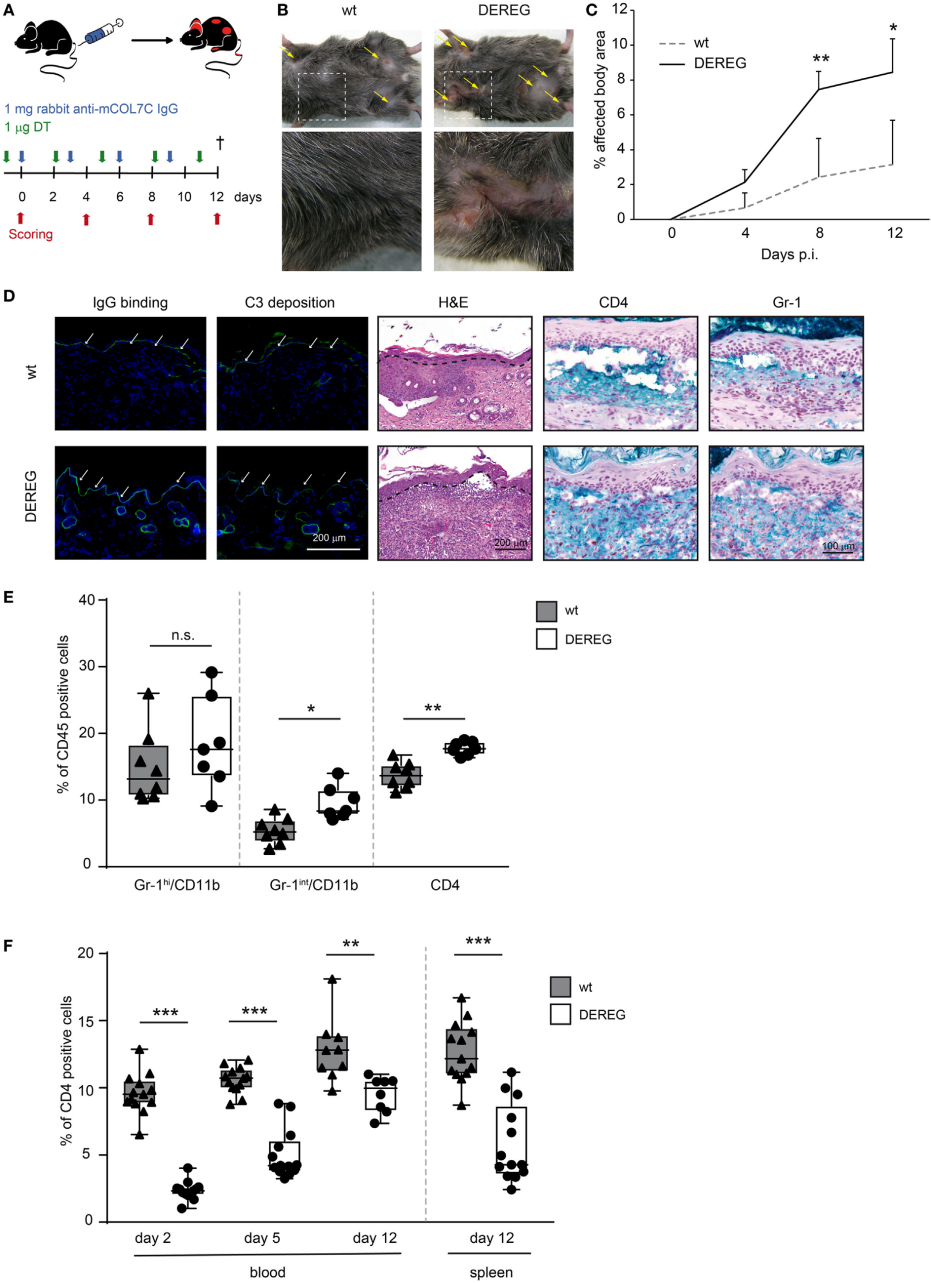
Depletion of regulatory T cells (Tregs) increases disease progression in experimental epidermolysis bullosa acquisita (EBA). **(A)** EBA was induced by repetitive injections of 4× 1 mg rabbit anti-mouse COL7 in wild-type or depletion of regulatory T cell (DEREG) mice, which were injected with diphtheria toxin (DT). **(B,C)** Treg depletion in DEREG mice led to a significant (more than twofold) increase in skin inflammation and blistering over a period of 12 days. Panel **(B)** shows representative clinical images obtained on day 12 of the experiment. Panel **(C)** displays the development of the affected body surface area over the 12-day observation period. **(D)** Mice after 12 days of experimental EBA were analyzed for IgG binding, C3 deposition, leukocyte dermal infiltration, and presence of CD4-positive T cells and Gr-1-positive cells (neutrophils and macrophages): staining with an anti-rabbit IgG-FITC antibody or anti-rabbit C3-FITC antibody respective showed no differences in IgG and C3 deposition at the DEJ. Histology (hematoxylin and eosin staining) indicated stronger inflammation in the epidermis and more split formation at the DEJ of DEREG ear sections; the DEJ is marked by a dotted line. Both wild-type and DEREG mice had strong infiltrates of CD4 T cells and Gr-1 (Fast Blue)-positive cells. **(E)** The amount of different CD45-positive populations (Gr-1^hi^/CD11b^pos^ macrophages, Gr-1^int^/CD11b^pos^ neutrophils, and CD4^pos^ T cells) was evaluated from lysed spleens at day 12 of experimental EBA or blood from days 2, 5, and 12. DEREG mice have increased numbers of CD4 T cells and Gr-1^int^/CD11b^pos^ neutrophils. **(F)** The efficacy of Treg depletion after DT treatment was evaluated from the same time points. DEREG mice have significantly reduced numbers of Foxp3-positive cells. **(C,E,F)** Mann–Whitney *U*-test with a Bonferroni *post hoc* test (**p < 0.01, ***p < 0.001): **(C)** the mean (+SD), *n* = 8/group **(E,F)**. The data are presented as medians (black line), 25th/75th percentiles (boxes), and max/min values (error bars); the dots represent actual results for each sample.

**Figure 2:**
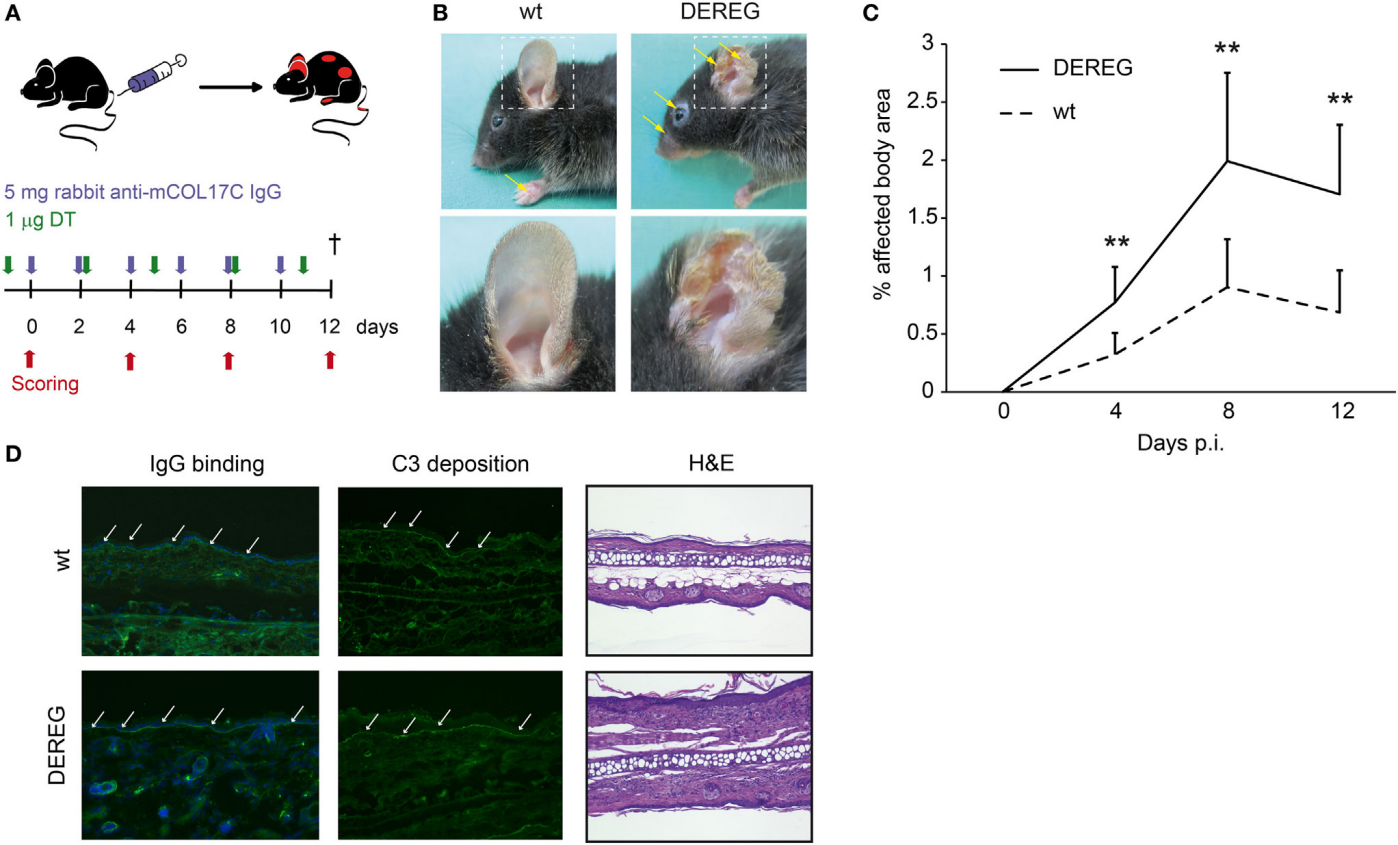
Depletion of regulatory T cells (Tregs) increases disease progression in experimental BP. **(A)** BP was induced by repetitive injections of 6× 5 mg rabbit anti-mouse COL17 in wild-type or DEREG mice, which were injected with diphtheria toxin (DT). **(B,C)** Treg depletion in DE REG mice led to a significant increase in disease progression. Panel **(B)** shows representative clinical images obtained on day 12 of the experiment. Panel **(C)** displays the development of the affected body surface area over the 12-day observation period. **(D)** Staining with anti-rabbit IgG-FITC antibody showed no differences in IgG deposition at the DEJ. Histology (hematoxylin and eosin staining) indicated increased inflammation in the epidermis of DEREG ear sections. **(C)** Mann–Whitney *U*-test (**p < 0.01): the mean (+SD), *n* = 9/group.

To investigate the impact of Tregs on skin inflammation and blistering in PDs, we assessed the impact on Treg depletion in antibody transfer models of BP (20) and EBA (26). In brief, experimental PD was induced by repetitive injections of rabbit anti-mouse COL7 (Figure 1A) or anti-mouse COL17 IgG (Figure 2A) in wild-type or DEREG mice (5), which were both injected with DT. Subsequently, clinical disease manifestation, expressed as body surface area affected by PD skin lesions, was assessed as the primary endpoint.

In antibody transfer-induced EBA, Treg depletion in DEREG mice led to a significant (more than twofold) increase in skin inflammation and blistering (Figure 1B,C), accompanied by an increase in leukocyte dermal infiltration, while IgG binding and C3 deposition at the dermal-epidermal junction were not affected (Figure 1D) compared with DT injected wild-type controls. In the dermal infiltrate, we could detect large numbers of Gr-1-positive cells (neutrophils and macrophages) and CD4-positive cells (Figure 1D). To verify whether these cell types are influenced by Treg depletion, we analyzed them in the spleens of DEREG and wild-type mice after 12 days of experimental EBA (Figure 1E). Here, in DEREG mice, the percentages of CD4 T cells and Gr-1^int^/CD11b neutrophils are significantly increased, but the number of Gr-1^hi^/CD11b macrophages is stable. These data implicate an effect of Treg depletion on CD4 T cells and neutrophils that will be investigated in more detail. Importantly, an injection of the same amount of normal rabbit IgG into DT-treated DEREG mice was not sufficient to induce a skin blistering phenotype, but an increase of Gr-1-positive cells in the dermis was detectable (Figure S1 in Supplementary Material). To control the depletory effect of the DT treatment on DEREG mice, we examined the number of Foxp3/CD25-positive CD4 T cells and showed that the depletion is valid throughout the whole experimental procedure in blood and spleen. The number of Tregs is reduced by half (Figure 1F).

Corresponding observations were made in antibody transfer-induced BP (Figure 2). Again, the depletion of Tregs in DEREG mice led to significant aggravation of skin inflammation and blistering (Figures 2A–C) and an increase in leukocyte dermal infiltration (Figure 2D), while IgG binding and C3 deposition (Figure 2D) at the dermal-epidermal junction were not affected. Taken together, we demonstrate the crucial role of Tregs in dampening (auto) antibody-induced, myeloid cell-driven skin inflammation and blistering.

### Depletion of Tregs Induces a Pro-inflammatory Cytokine Milieu in Skin and Serum during PD

**Figure 3:**
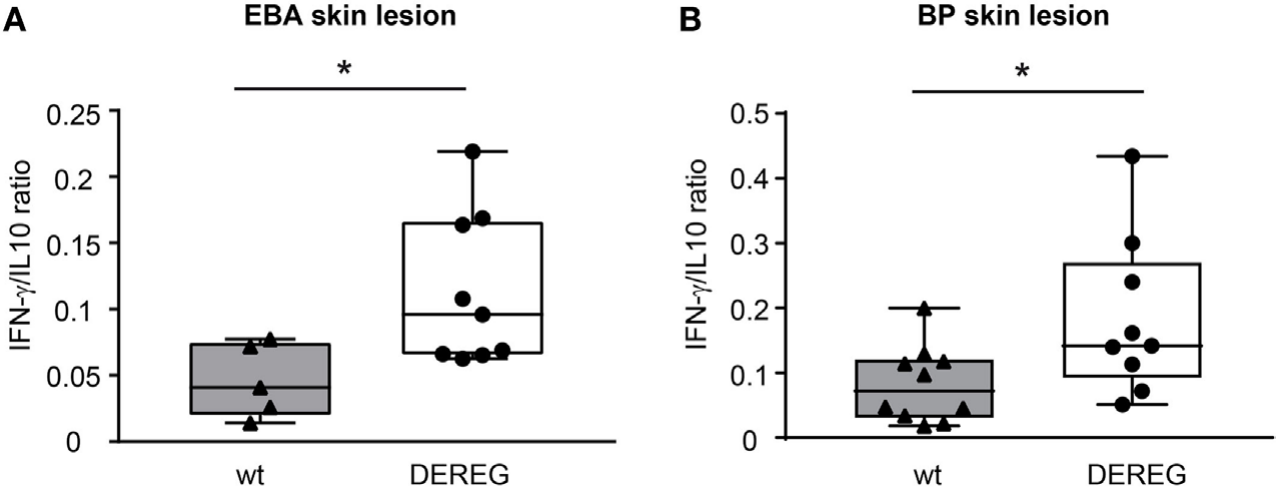
Depletion of regulatory T cells increases Th1/Th2 ratio in depletion of regulatory T cell (DEREG) mice. **(A)** Epidermolysis bullosa acquisita (EBA) was induced by repetitive injections of 4× 1 mg rabbit anti-mouse COL7 in wild-type (wt) or DEREG mice, which were injected with diphtheria toxin (DT). The mRNA expression ratio of Th1-specific *IFN-*γ to Th2-specific *IL-10* is significantly shifted to a more Th1-specific phenotype in the skin. **(B)** BP was induced by repetitive injections of 6× 5 mg rabbit anti-mouse COL17 in wild-type or DEREG mice, which were injected with DT. Mann–Whitney *U*-test (*p < 0.05), *n* = 8–9/group.

In principle, Tregs could modulate myeloid-driven skin inflammation by two mechanisms that are not mutually exclusive. First, Tregs could alter the migratory behavior of myeloid cells. Second, Tregs might dampen IC-induced myeloid cell activation. Both myeloid migration and activation are essential for inflammation and blistering in PD (12). To address the first possibility, chemokine and cytokine expression in lesional skin of wild-type or DEREG mice after experimental BP and EBA was evaluated (for comparison to healthy skin see Table S1 in Supplementary Material). Interestingly, the expression of innate cytokines, such as *TNF*, known as prominent cytokines in PD skin lesions (13, 27), did only slightly differ between wild-type and DEREG mice and was not significant in BP skin lesions (Tables 1 and 2). Of note, the Th2 cytokines *IL-4, IL-10*, and *IL-13* were significantly higher expressed in lesional DEREG skin (Tables 1 and 2). The presence of these Th2 cytokines would implicate a rather anti-inflammatory cytokine milieu. By contrast, the expression of the inflammatory Th1 cytokine *IFN-*γ and the chemokine *CXCL-9* was evaluated in DEREG lesions. Therefore, the increased dermal infiltrate observed in DEREG mice after PD induction is most likely driven by *IFN-*γ and *CXCL-9* (28, 29), whereas the anti-inflammatory properties of the other differentially expressed cytokines, especially *IL-10* (30), are not sufficient to prevent blistering. The ratio of Th2-specific *IL-10* to Th1-specific *IFN-*γ is significantly shifted to a more Th1-specific phenotype in DEREG mice in EBA and BP (Figure 3). In accordance with this finding, we evaluated pro-inflammatory cytokine expression in the serum of wild-type or DEREG mice after 12 days of PD; here, the most prominent cytokine is IFN-γ, which is released in DEREG mice but nearly undetectable in wild-type mice in experimental EBA and BP (Tables 3 and 4). In addition, in experimental BP IL-1β that is slightly reduced. By contrast, IL-10 shows no significant change in the serum, which is in line with the assumption that the increased expression of anti-inflammatory cytokines in the skin is ineffective.

**Table 1:** Analysis of mRNA in skin biopsies of wt or depletion of regulatory T cell (DEREG) mice after experimental epidermolysis bullosa acquisita.

mRNA	wt	DEREG	*p*-Value
*Cd3*	0.00041 ± 0.00045	0.00207 ± 0.00297	0.027
*Cd19*	0.00000 ± 0.00000	0.00000 ± 0.00000	> 0.999
*Gr-1*	0.01769 ± 0.01580	0.03129 ± 0.03653	0.626
*Cd11c*	0.03212 ± 0.02166	0.03428 ± 0.02354	0.912
*Mac-1*	0.28203 ± 0.27184	0.40627 ± 0.31225	0.353
*Il-10*	0.00055 ± 0.00102	0.00115 ± 0.00088	0.033
*Tnf-*α	0.01174 ± 0.01139	0.01492 ± 0.01259	0.033
*Il-17A*	0.00270 ± 0.00680	0.00240 ± 0.00343	0.436
*Ifn-*γ	0.00001 ± 0.00001	0.00011 ± 0.00005	0.001
*Il-4*	0.00017 ± 0.00016	0.00104 ± 0.00108	0.001
*Il-33*	0.39346 ± 0.39972	0.31200 ± 0.38451	0.393
*Cxcl-2/MIP-2*	2.95181 ± 2.44523	2.76091 ± 3.20865	0.9182
*CCR5*	0.15055 ± 0.13897	0.26642 ± 0.29714	0.594
*CCR7*	0.01631 ± 0.02362	0.02631 ± 0.04100	0.620
*Il-13*	0.00004 ± 0.00004	0.00026 ± 0.00034	0.009
*Cxcl-1/KC*	0.01131 ± 0.01117	0.04612 ± 0.06437	0.075
*Cxcl-9/MIG*	0.01325 ± 0.03193	0.02061 ± 0.01402	0.013
*Cxcl-10/IP-10*	0.01403 ± 0.03168	0.00873 ± 0.00451	0.393

*DEREG and wild- type (wt) mice were injected with 4× 1 mg rabbit anti-mCOL7 IgG, and lesional skin (of comparable disease index) was taken for mRNA extraction. Analysis of mRNA by qRT-PCR for the indicated markers was done relative to the housekeeping gene GAPDH using the 2^ΔCT^ method. Mann–Whitney *U*-test, mean (±SD), *n* = 9/group. Significant differences in gene expression are indicated in gray*.

**Table 2:** Analysis of mRNA in skin biopsies of wt or depletion of regulatory T cell (DEREG) mice after experimental pemphigoid disease.

mRNA	wt	DEREG	*p*-Value
*Cd3*	0.00216 ± 0.00368	0.00490 ± 0.00389	0.123
*Cd19*	0.00003 ± 0.00007	0.00001 ± 0.00001	0.283
*Gr-1*	0.00104 ± 0.00106	0.00156 ± 0.00201	0.483
*Cd11c*	0.03002 ± 0.02234	0.03024 ± 0.01847	0.981
*Mac-1*	0.08454 ± 0.05235	0.15347 ± 0.10119	0.072
*Il-10*	0.00066 ± 0.00044	0.00131 ± 0.00082	0.039
*Tnf-*α	0.00593 ± 0.00428	0.00895 ± 0.00759	0.288
*Il-17A*	0.00000 ± 0.00001	0.00001 ± 0.00001	0.413
*Ifn-*γ	0.00006 ± 0.00006	0.00017 ± 0.00010	0.008
*Il-4*	0.00048 ± 0.00034	0.00214 ± 0.00184	0.011
*Il-33*	0.26453 ± 0.19083	0.18644 ± 0.10926	0.276
*CXCL-2/MIP-2*	0.99754 ± 0.91675	0.97397 ± 1.15808	0.960
*Ccr5*	0.17625 ± 0.20268	0.26582 ± 0.20309	0.337
*Ccr7*	0.01070 ± 0.00833	0.00983 ± 0.00708	0.804
*Il-13*	0.00012 ± 0.00008	0.00027 ± 0.00015	0.014
*Cxcl-1/KC*	0.00397 ± 0.00531	0.00360 ± 0.00291	0.849
*Cxcl-9/MIG*	0.01409 ± 0.01044	0.04539 ± 0.03481	0.014
*Cxcl-10/IP-10*	0.01556 ± 0.01446	0.01280 ± 0.00842	0.608

*DEREG and wild-type (wt) mice were injected with 6× 5 mg rabbit anti-mCOL17 IgG at days 0, 2, 4, 6, 8, and 10, and lesional skin (of comparable disease index) was taken for mRNA extraction. Analysis of mRNA by qRT-PCR for the indicated markers was done relative to the housekeeping gene GAPDH using the 2^ΔCT^ method. Mann–Whitney *U*-test, mean (±SD), *n* = 10/group. Significant differences in gene expression are indicated in gray*.

**Table 3:** Analysis of inflammatory cytokines in serum of wt or depletion of regulatory T cell (DEREG) mice after experimental epidermolysis bullosa acquisita.

Cytokine	wt	DEREG	*p*-Value
IL-1α	32.50 ± 23.97	25.53 ± 14.97	0.734
IL-23	97.29 ± 86.81	160.32 ± 143.70	0.379
IFN-γ	4.74 ± 4.07	11.32 ± 6.63	0.010
TNF-α	8.60 ± 7.45	12.36 ± 6.17	0.179
IFN-β	327.43 ± 257.70	381.63 ± 289.79	0.878
GM-CSF	67.56 ± 42.99	83.90 ± 41.87	0.403
CCL2/MCP-1	26.80 ± 27.20	46.06 ± 25.70	0.079
IL-1β	36.08 ± 31.64	44.12 ± 28.47	0.516
IL-10	102.49 ± 84.85	135.66 ± 120.32	0.647
IL-12p70	1.31 ± 2.71	4.30 ± 5.76	0.189
IL-6	36.66 ± 25.52	44.77 ± 30.62	0.600
IL-27	544.88 ± 370.61	594.16 ± 383.73	0.830
IL-17A	28.84 ± 16.32	43.14 ± 30.56	0.225

*DEREG and wild-type (wt) mice were injected with rabbit anti-mCOL7 IgG, and serum was taken for LEGENDplex™ cytokine analysis. Values are indicated as picograms per milliliter serum. Mann–Whitney *U*-test, mean (±SD), *n* ≥ 8/group. Outliners were excluded from analysis. Significant differences in cytokine concentrations are indicated in gray*.

**Table 4:** Analysis of inflammatory cytokines in serum of wt or depletion of regulatory T cell (DEREG) mice after experimental pemphigoid disease.

Cytokine	wt	DEREG	*p*-Value
IL-1α	5.48 ± 5.81	10.48 ± 8.83	0.076
IL-23	7.52 ± 8.86	7.79 ± 13.84	0.479
IFN-γ	0.02 ± 0.07	1.27 ± 1.11	0.001
TNF-α	2.42 ± 3.57	8.03 ± 18.69	0.182
IFN-β	44.89 ± 45.57	85.42 ± 110.01	0.148
GM-CSF	10.37 ± 15.39	8.21 ± 11.06	0.362
CCL2/MCP-1	0.42 ± 0.73	0.23 ± 0.53	0.258
IL-1β	1.66 ± 2.77	0.00 ± 0.00	0.037
IL-10	12.83 ± 13.90	9.65 ± 14.54	0.311
IL-12p70	0.42 ± 0.73	0.23 ± 0.53	0.258
IL-6	3.44 ± 4.72	152.56 ± 476.70	0.168
IL-27	20.21 ± 15.36	13.68 ± 18.04	0.197
IL-17A	1.38 ± 1.53	0.48 ± 0.83	0.060

*DEREG and wild-type (wt) mice were injected with 6× 5 mg rabbit anti-mCOL17 IgG at days 0, 2, 4, 6, 8, and 10, and serum was taken for LEGENDplex™ cytokine analysis. Values are indicated as picograms per milliliter serum. Mann–Whitney *U*-test, mean (±SD), *n* = 10/group. Significant differences in cytokine concentrations are indicated in gray*.

### Coculture of Tregs with PMNs Changes the Surface Expression of Integrins after IC Stimulation

**Figure 4:**
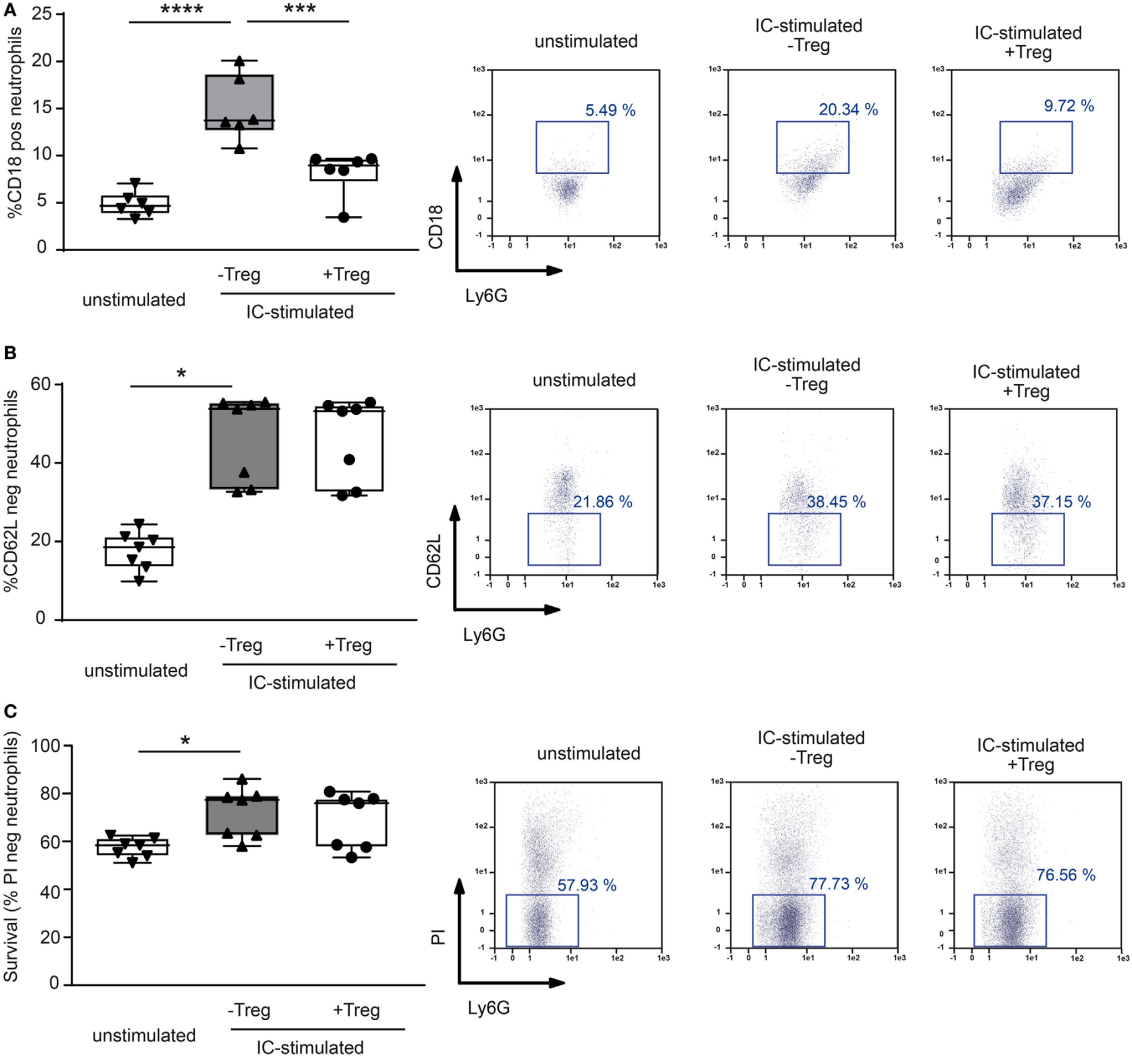
Coculture of regulatory T cells (Tregs) and PMNs after immune complex (IC) stimulation changes surface marker expression in neutrophils. Freshly isolated murine PMNs were stimulated in presence of mCOL7/anti-mCOL7 ICs for 1 h. Tregs were added for additional 4.5 h, and the surface expression of **(A)** CD18 and **(B)** CD62L expression was evaluated on CD45^pos^/Ly6G^pos^/PI^neg^ neutrophils. Stimulation with ICs strongly increased the expression of CD18 and shedding of CD62L, but addition of Tregs inhibited only CD18 expression. **(C)** The survival of IC-stimulated PMNs was not affected by Tregs as indicated by measurement of propidium iodide (PI) negative CD45^pos^/Ly6G^pos^ cells. One-way ANOVA test with a Bonferroni *post hoc* test (*p < 0.05): the data are presented as medians (black line), 25th/75th percentiles (boxes), and max/min values (error bars). The dots represent actual results for each sample (*n* = 7–8/group).

**Figure 5:**
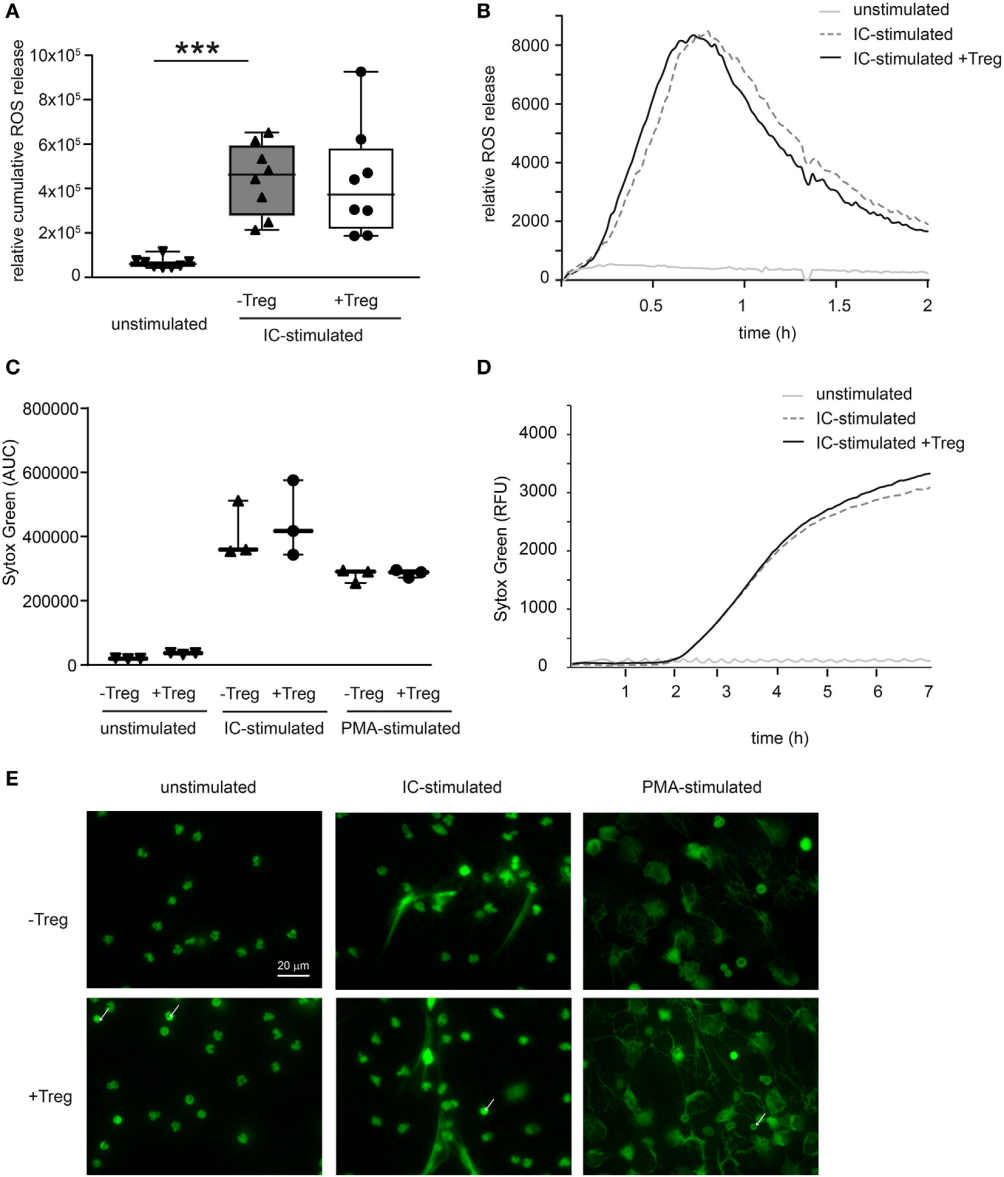
Coculture of regulatory T cells (Tregs) and PMNs after immune complex (IC) stimulation does not affect reactive oxygen species (ROS) release and neutrophil extracellular trap (NET) formation. **(A)** Freshly isolated murine PMNs were pre-cultured with freshly isolated Tregs for 1 h followed by IC stimulation with addition of luminol stimulated in presence of mCOL7/anti-mCOL7 ICs for 2 h. Relative ROS release is indicated by chemiluminescence. Tregs have no influence on ROS release in PMNs. **(B)** Representative real-time kinetics of ROS release using mCOL7/anti-mCOL7 IC. **(C)** Human neutrophils were preincubated for 30 min under HEPES-buffered conditions at pH 7.4 and were then stimulated for 4 h with PMA, for 7 h with mCOL7/anti-mCOL7 IC, or left untreated w/o human Treg to induce NET formation. Area under the curve values (mean ± SD) of NET-dependent relative fluorescence intensities as measured by the SYTOXgreen assay and **(D)** representative real-time kinetics of NET release measured by staining with SYTOXgreen. **(E)** For fluorescence microscopy, cells were fixed and stained for DNA by using SYTOXgreen (green) Treg have no effect on NET formation. One-way ANOVA test with a Bonferroni *post hoc* test (***p < 0.001): the data are presented as medians (black line), 25th/75th percentiles (boxes), and max/min values (error bars). The dots represent actual results for each sample (*n* = 3–8/group).

To investigate a potential direct effect of Tregs on PMN function in more detail, we performed *in vitro* assays and stimulated PMNs with ICs consisting of COL7/anti-COL7 in the presence or absence of Tregs (Figure 4). Whereas the expression of β2 integrin (CD18) is strongly reduced by Tregs (Figure 4A), the shedding of CD62L in Ly6G-positive neutrophils is unchanged (Figure 4B). This finding is in accordance with previous data that clearly show the importance of CD18 in experimental PD (31). An effect on the survival of PMNs in the observed time period could not be detected (Figure 4C). In contrast to the effects on the cell surface marker expression, Tregs do not influence the IC-induced ROS release of PMNs after 1 h preincubation of both cell types (Figures 5A,B) and had no effect on the production of NETs, which are released during a programmed cell death, the so-called NETosis induced by IC or PMA (Figures 5C–E).

## Discussion

**Figure 6:**
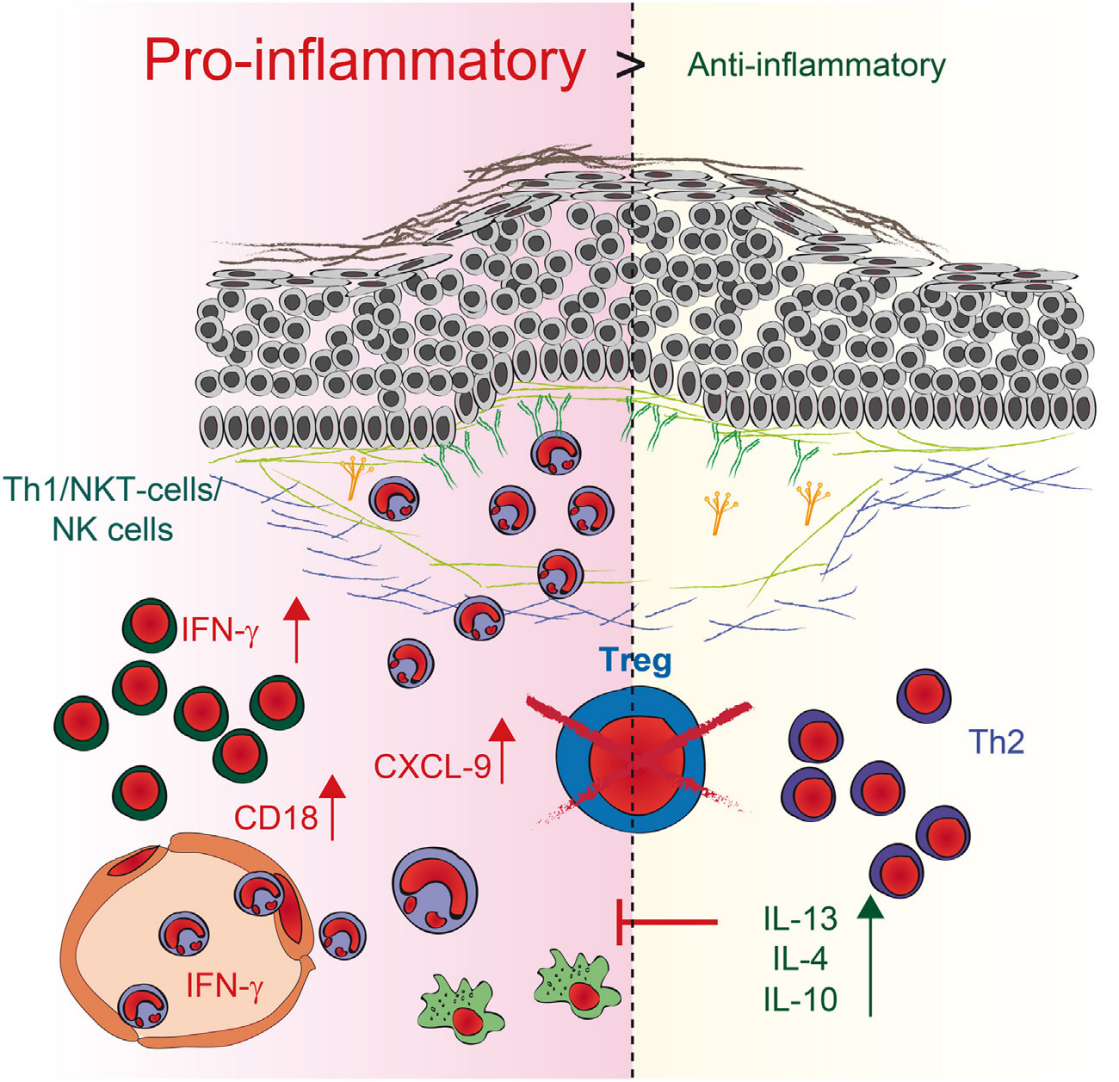
Schematic illustration of regulatory T cell (Treg) action during pemphigoid disease. Reduction of Tregs in depletion of regulatory T cell mice induces two independent pathways. First, the expression of anti-inflammatory IL-10- and Th2-specific cytokines, such as IL-13 and IL-4, is enhanced in the skin. Second, higher expression of the pro-inflammatory Th1 cytokine IFN-γ as well as the chemoattractant CXCL9 is detectable in the skin, accompanied by an increase of IFN-γ in the serum. The number of neutrophils in the blood and the amount of infiltration increase. Direct interaction of Tregs with neutrophils also blocks CD18 expression on neutrophils, indicating that an increase in CD18 after Treg depletion could be a possible mechanism by which more neutrophils infiltrate the skin. By contrast, the increase in Th2 cytokines may be a rather ineffective counter mechanism induced by the absence of Tregs.

Regulatory T cells are essential for establishing and maintaining self-tolerance and inhibiting immune responses to innocuous environmental antigens. Imbalances and dysfunction in Tregs lead to a variety of immune-mediated diseases because deficits in Treg function contribute to the development of autoimmune diseases and pathological tissue damage, whereas an overabundance of Tregs can promote chronic infection and tumorigenesis (4). To determine by what mechanism Tregs can contribute to the development of PDs, we used antibody transfer-induced models of experimental EBA and BP. By using DEREG mice, we depleted Tregs in these models and observed a twofold increase in clinical disease severity. Analysis of skin and serum of these mice, along with *in vitro* coculture experiments, revealed a dual mechanism by which Tregs can influence IC-induced inflammation in the skin (Figure 6). First, the mRNA expression of anti-inflammatory *IL-10*- and Th2-specific cytokines, such as *IL-13, IL-4*, and *IL-10*, is increased in the skin. These data are in accordance with the scurfy mouse phenotype, where the main effector cells are IL-6-, IL-10-, and IL-4-positive CD4 T cells (32). Under pathological conditions in the skin, these cells can contribute to allergic reactions and atopic dermatitis (33–36). Interestingly, no correlation with autoimmunity has been described thus far, indicating a possible insufficient counter mechanism, as described for IL-6 (19), where Tregs may self-inhibit their function in antibody-induced inflammation. Therefore, the anti-inflammatory properties of the other differentially expressed cytokines, especially IL-10 (30), are not sufficient to prevent blistering. Second, in addition to the fact that Th2 cytokines are increasingly expressed in the skin, the *IFN-*γ is strongly upregulated in the skin. IFN-γ, is secreted predominantly by T cells and natural killer (NK) cells (37) and, to a lesser extent, by other cell types such as macrophages, dendritic cells (DC) and B cells (38). It has been described, that during innate immune responses IFN-γ is produced by NK and NKT cells as well as macrophages and DCs whereas in adaptive immunity it is produced by CD8^+^ cells and Th1 cells (39). IFN-γ was linked to autoimmunity as upregulation is found in patients with different autoimmune diseases like systemic lupus erythematosus, Sjögren’s syndrome, polymyositis, dermatomyositis, and systemic sclerosis (39, 40). Fontolizumab, a humanized monoclonal antibody against IFN-γ, was well tolerated and showed some efficacy in patients with Crohn’s disease (41, 42). In this context, we could show that upregulation of *IFN-*γ in the skin, accompanied by an increase of IFN-γ in the serum after blocking Treg could be an important mechanism by which Treg normally contribute to the inhibition of immune responses. Together with the fact that inhibition of Treg led to an increase of the T cell chemoattractant *CXCL-9* in the skin this could subsequently increase the number of neutrophils in the blood and the amount of infiltrating cells into the inflamed skin.

In addition to the effect on T cells, the absence of Tregs in DEREG mice also directly affect the number of neutrophils within the dermal infiltrate in PDs. Direct interaction of Tregs with neutrophils also blocks CD18 expression on neutrophils, indicating that an increase in CD18 after Treg depletion could be a possible mechanism to attract more neutrophil infiltrates in the skin. The fact that Tregs block CD18 expression in IC-stimulated neutrophils is in accordance with previous data where CD18-deficient mice were resistant to experimental EBA due to defective recruitment of PMNs into the inflamed dermis (31, 43). In addition, previous publications using a mouse melanoma model described that Tregs limit neutrophil accumulation and survival and thus, promoted survival of the inoculated tumor cells. This effect was associated with decreased expression of the neutrophil chemoattractants CXCL1 and CXCL2 (7). Further *in vitro* coculture studies of LPS-stimulated human Tregs and neutrophils demonstrated a decrease in CD62L shedding after 45 min of incubation and a decrease in IL-6, IL-8, and TNF-α production after 18 h of incubation. Neutrophil death was doubled in the presence of Tregs that had been stimulated with LPS (8). In our hands, the coculture was performed using IC-stimulated neutrophils rather than LPS-activated neutrophils. During 4.5 h of incubation, ICs induced a significant increase in neutrophil cell death, but this observation was independent from Tregs. Still, it remains unclear whether the influence of Treg on IC-activated neutrophils is mediated by direct cell–cell contact or by Treg cytokines but earlier studies showed a possible effect of IL-10 on neutrophils (7).

These findings, however, do not allow to distinguish between cause and effect; i.e., the altered cytokine expression levels in DEREG mice may be either due to the absence of Tregs or they could merely reflect the degree of disease severity. Although, since in this study we compared the cytokine concentrations in lesional skin, the differential expression of cytokines is likely caused by the absence or presence of Tregs. Yet, further studies are needed to fully unravel the mechanisms by which Treg contribute to the inflammatory processes in PD. In addition, it is unclear whether these mechanisms are completely overlapping in humans and mice. It has been shown that the number of Tregs increase in mice and man under inflammatory conditions (44). In humans, specialized populations of Treg cells may be recruited to different types of inflammatory responses, and it has been discussed that these may share molecular characteristics with pro-inflammatory helper T cell populations (4). Therefore, in humans, the Foxp3-positive cells are more heterogeneous in function than mouse Foxp3-positive cells. This fact makes it difficult to use autologous Tregs transplantation as therapeutic targets (45).

In summary, we demonstrated that Tregs control myeloid cell-mediated skin inflammation in experimental PD. Furthermore, our data suggest this novel property of Tregs to be mediated by the modulation of cytokine production and by a change in the expression of neutrophil surface markers in the skin, thus regulating leukocyte extravasation into the skin.

## Ethics Statement

This study was carried out in accordance with the recommendations of European Community rules for animal care. The protocol was approved by the Ministry for Energy, Agriculture, the Environment and Rural Areas, Kiel, Schleswig-Holstein, Germany. This study was carried out in accordance with the recommendations of the Declaration of Helsinki by the Ethical Committee of the Medical Faculty of the University of Lübeck with written informed consent from all subjects. All subjects gave written informed consent in accordance with the Declaration of Helsinki. The protocol was approved by the Ethical Committee of the Medical Faculty of the University of Lübeck.

## Author Contributions

KB and RL designed the research and wrote the manuscript. KB, SS, MW, AK, MB, FB, FS, NR, and RP performed experiments and analyzed data. KB, DZ, ES, EP, TL, TS, KK, and RL analyzed data and discussed the results. All the authors drafted the work critically and approved the final version. All the authors agreed to be accountable for all aspects of the work in ensuring that questions related to the accuracy or integrity of any part of the work are appropriately investigated and resolved.

## Conflict of Interest Statement

The authors declare that the research was conducted in the absence of any commercial or financial relationships that could be construed as a potential conflict of interest.
