# Supplementary material for: Regulatory T Cells Suppress Inflammation and Blistering in Pemphigoid Diseases
Source: Front Immunol. 2017 Nov 24;8:1628. doi: 10.3389/fimmu.2017.01628 (PMC5705561; doi:10.3389/fimmu.2017.01628)
Supplement: Supplementary file 1 [file table_1.docx]

| mRNA | healthy | | | *p*-Value vs. EBA | *p*-Value vs. BP |
| --- | --- | --- | --- | --- | --- |
| *Cd3* | 0.00016 | ± | 0.00010 | 0.304 | 0.020 |
| *Cd19* | 0.00000 | ± | 0.00000 | 0.435 | 0.408 |
| *Gr-1* | 0.00025 | ± | 0.00036 | 0.038 | 0.139 |
| *Cd11c* | 0.10593 | ± | 0.11411 | 0.055 | 0.055 |
| *Mac-1* | 0.05525 | ± | 0.03325 | 0.040 | 0.242 |
| *Il-10* | 0.00003 | ± | 0.00006 | 0.204 | 0.004 |
| *Tnf-α* | 0.00059 | ± | 0.00042 | 0.975 | 0.009 |
| *Il-17A* | 0.00236 | ± | 0.00025 | 0.871 | <0.001 |
| *Ifn-*γ | 0.00000 | ± | 0.00000 | 0.029 | 0.038 |
| *Il-4* | 0.00007 | ± | 0.00007 | 0.249 | 0.026 |
| *Il-33* | 0.10020 | ± | 0.06400 | 0.108 | 0.116 |
| *Cxcl-2 / MIP-2* | 0.04527 | ± | 0.04316 | 0.013 | 0.042 |
| *CCR5* | 0.24393 | ± | 0.14572 | 0.074 | 0.314 |
| *CCR7* | 0.00286 | ± | 0.00142 | 0.037 | 0.070 |
| *Il-13* | 0.00002 | ± | 0.00005 | 0.473 | 0.023 |
| *Cxcl-1 / KC* | 0.00124 | ± | 0.00123 | 0.048 | 0.326 |
| *Cxcl-9 / MIG* | 0.00467 | ± | 0.00357 | 0.677 | 0.053 |
| *Cxcl-10 / IP-10* | 0.00135 | ± | 0.00142 | 0.177 | 0.033 |
