# Supplementary figures and images for: Regulatory T Cells Suppress Inflammation and Blistering in Pemphigoid Diseases
Source: Front Immunol. 2017 Nov 24;8:1628. doi: 10.3389/fimmu.2017.01628 (PMC5705561; doi:10.3389/fimmu.2017.01628)

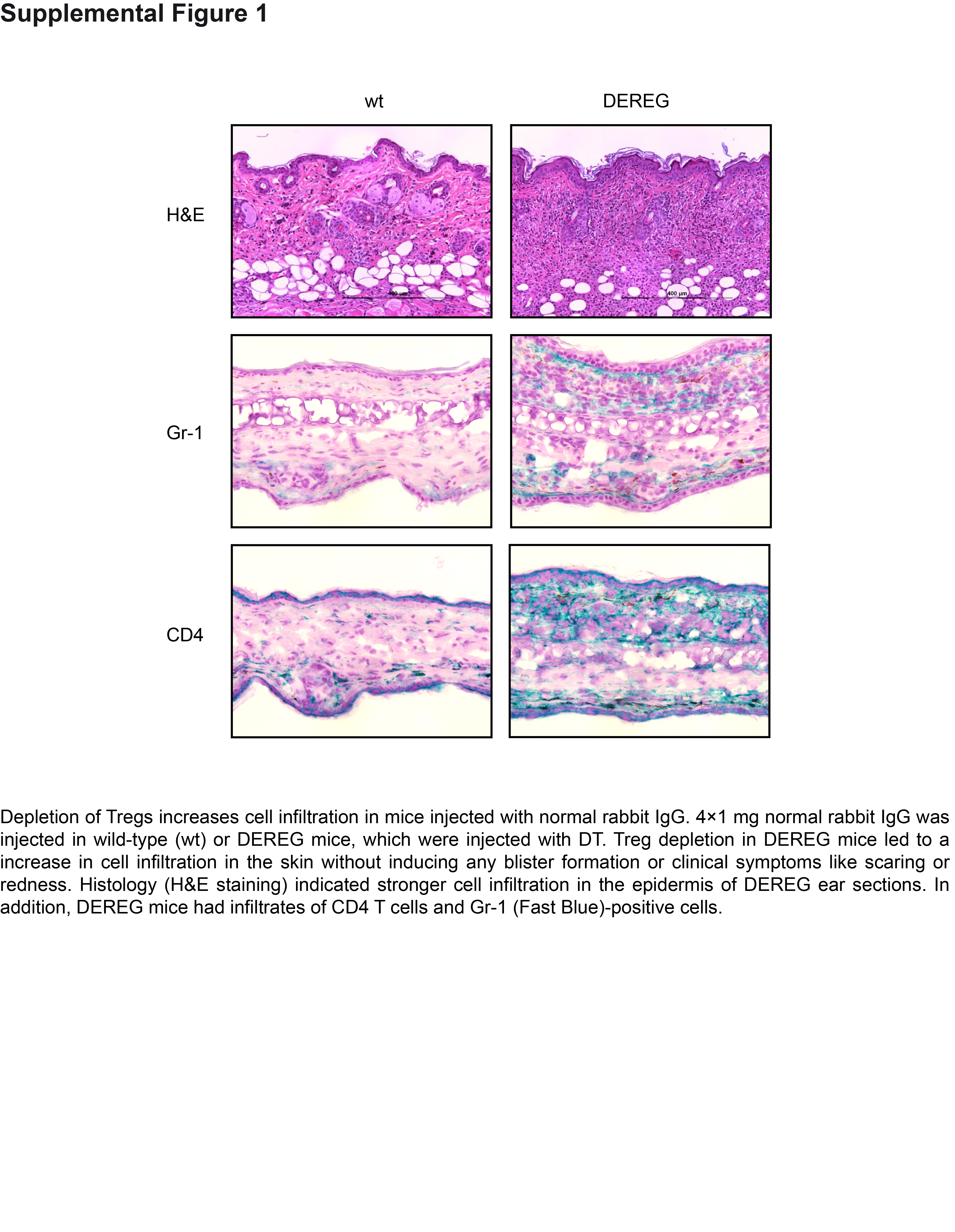

Supplement: Supplementary file 2 [file image_1.jpeg]
